# Implementation of Wavelet-Transform-Based Algorithms in an FPGA for Heart Rate and RT Interval Automatic Measurements in Real Time: Application in a Long-Term Ambulatory Electrocardiogram Monitor

**DOI:** 10.3390/mi14091748

**Published:** 2023-09-07

**Authors:** José Alberto García Limón, Frank Martínez-Suárez, Carlos Alvarado-Serrano

**Affiliations:** Bioelectronics Section, Department of Electrical Engineering, Centro de Investigación y de Estudios Avanzados del Instituto Politécnico Nacional (CINVESTAV), Mexico City 07360, Mexico; josea.garcial@cinvestav.mx (J.A.G.L.); frank.martinez@cinvestav.mx (F.M.-S.)

**Keywords:** ECG, Holter monitor, heart rate, RT interval, wavelet transform, FPGA

## Abstract

Cardiovascular diseases are currently the leading cause of death worldwide. Thus, there is a need for non-invasive ambulatory (Holter) ECG monitors with automatic measurements of ECG intervals to evaluate electrocardiographic abnormalities of patients with cardiac diseases. This work presents the implementation of algorithms in an FPGA for beat-to-beat heart rate and RT interval measurements based on the continuous wavelet transform (CWT) with splines for a prototype of an ambulatory ECG monitor of three leads. The prototype’s main elements are an analog–digital converter ADS1294, an FPGA of Xilinx XC7A35T-ICPG236C of the Artix-7 family of low consumption, immersed in a low-scale Cmod-A7 development card integration, an LCD display and a micro-SD memory of 16 Gb. A main state machine initializes and manages the simultaneous acquisition of three leads from the ADS1294 and filters the signals using a FIR filter. The algorithm based on the CWT with splines detects the QRS complex (R or S wave) and then the T-wave end using a search window. Finally, the heart rate (60/RR interval) and the RT interval (from R peak to T-wave end) are calculated for analysis of its dynamics. The micro-SD memory stores the three leads and the RR and RT intervals, and an LCD screen displays the beat-to-beat values of heart rate, RT interval and the electrode connection. The algorithm implemented on the FPGA achieved satisfactory results in detecting different morphologies of QRS complexes and T wave in real time for the analysis of heart rate and RT interval dynamics.

## 1. Introduction

Cardiovascular diseases have remained the leading cause of death globally and considerably contribute to loss of health and excess health system costs [1,2]. Therefore, the development of ambulatory (Holter) electrocardiogram (ECG) monitors that allow the non-invasive continuous evaluation of abnormal cardiac activity during normal daily activities, and additionally perform beat-to-beat heart rate and real-time ECG interval measurements of electrocardiographic parameters is important in clinical practice for the diagnosis, prognosis and treatment of cardiovascular diseases.

**Figure 1:**
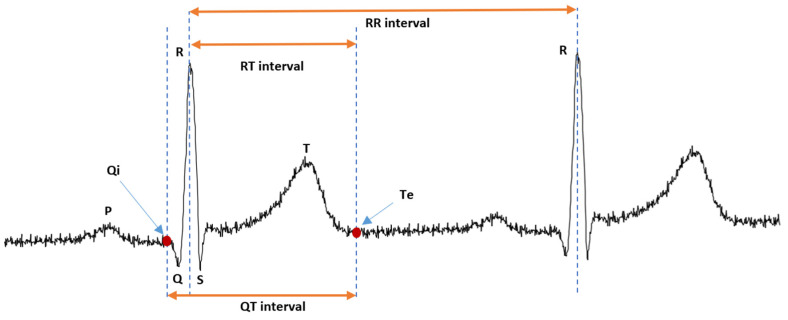
ECG signal, its waves and the three characteristic intervals RR, QT and RT.

The ECG is the recording of the electrical activity of the heart measured between two points on the surface of the body. In Figure 1, waves and intervals of the ECG are shown. The P, QRS and T waves reflect the rhythmic electrical depolarization and repolarization of the myocardium associated with the contractions of the atria and ventricles. The P wave represents depolarization of the atrial musculature. The QRS complex is the combined result of the repolarization of the atria and the depolarization of the ventricles, which occur almost simultaneously. The T wave represents ventricular repolarization. Time intervals are important in electrocardiographic diagnosis because they reflect electrophysiological processes; hence, under pathological conditions they have durations outside the range of the normal variation [3]. The RR interval, measured from the R wave peak ® to the peak of the next consecutive R wave, is the interval between consecutive heart beats and determines heart rate. The QT interval, measured from the Q wave onset (Qi) to the T wave end (Te), reflects the total period of ventricular depolarization and repolarization, and is used in clinical electrocardiology to quantify the duration of ventricular repolarization. RT interval (measured from R to Te) has been used instead of the QT interval to calculate its variability, and it can be used as an approximation of QT interval [4].

Some parameters that can be measured with the long-term ECG recordings of the Holter monitor are the repolarization dynamics and variability, which reflect changes in myocardial vulnerability and contribute to increased risk of arrhythmic events and sudden death. Repolarization dynamics is defined as a phenomenon that describes and quantifies QT adaptation to changing heart rate and is evaluated with the QT-RR slopes. QT variability reflects beat-to-beat changes in repolarization duration and morphology [5]. Another parameter considered as a risk predictor of cardiovascular death and arrhythmic events that can be measured is the RR interval changes between consecutive beats for the analysis of heart rate variability (HRV) [6].

Thanks to technological advances, Holter monitors have also evolved in terms of signal processing, storage, size and weight [7]. The processes of ECG signal acquisition, parameter extraction, storage and display of the information in real time can have a high computational cost depending on the number of leads obtained and the algorithm used. Because of this, have FPGAs emerged as a powerful tool since their architecture allows parallel processing, which makes them ideal for processing multiple ECG leads.

There is a wide variety of works in which an FPGA is used as a central processing system for QRS complex detection to obtain the heart rate, as well as for the classification of arrhythmias. Among the detection techniques used are the integer Haar transform [8], wavelet transform [9,10], the centered derivative and the intermediate value theorem [11], adaptative threshold [12], Shannon energy envelope [13], Pan–Tompkins algorithm [14] and a modified version [15]. The continuous wavelet transform (CWT) is an effective method for the analysis of non-stationary signals and allows the identification of different frequency events within a defined bandwidth (e.g., the QRS complex), which makes it ideal for ECG processing [16]. The advantages of wavelet transform compared to other techniques include its ability to perform time-frequency analysis, which provides information regarding three dimensions of the ECG signal: time, frequency and amplitude. As the frequency content of the ECG waves varies in time (QRS complex is a high-frequency wave and T wave is a low-frequency wave), utilization of this technique with different scales (bandwidths) allows an accurate time location and frequency identification of the characteristic points of ECG waves and reduces the effects of noise, baseline drifts and artifacts in the ECG signal [17,18]. 

In this work, we present an extended version of the conference paper published in [19], in which we showed the development of a prototype of an ambulatory long-term ECG monitor based on FPGA for the simultaneous acquisition and storage of three leads, without detailing the real-time detection algorithm of the ECG points used. Therefore, the aim of this paper is to describe in depth the implementation process of the CWT with splines in an FPGA, and its application in the beat-to-beat detection of the ECG points QRS and Te for the measurements of heart rate and RT interval in real time.

## 2. Materials and Methods

According to our previous work [19], the Holter prototype consists of 3 fundamental elements:

- Xilinx Cmod A7 development board, which contains a low power consumption Artix-7 XC7A35T-ICPG236C family FPGA.
- Texas Instruments ADS1294 integrated circuit with low power consumption, 4 channels, 24-bit analog-to-digital converters and ECG acquisition module.
- 16 GB Kingston Class 10 micro SDHC memory.

### 2.1. Software Implemented

**Figure 2:**
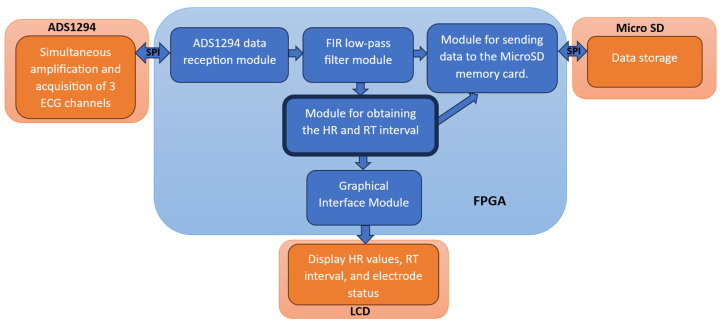
Modules implemented in the FPGA and their interaction with different peripherals.

To control each of the peripherals used, specific modules in the FPGA were developed to ensure proper synchronization (see Figure 2). The first module initializes and receives the signal from 3 channels of ECG from the ADS1294 acquisition circuit. Next, the following module receives the data from the 3 channels of ECG and applies a low-pass FIR filtering to limit the bandwidth to 200 Hz. The filtered signals are sent both to the storage module and to the module for obtaining the parameters of heart rate (HR) and RT interval based on CWT with splines. These two parameters are also stored and sent to a module for display on an LCD screen. To achieve optimal synchronization among the various peripherals and modules implemented in the FPGA, a general state machine is responsible for managing the device’s operation, maintaining an orderly sequence of operation for each of the modules ([19], Figure 2).

#### 2.1.1. Continuous Wavelet Transform

The wavelet transform is extensively employed for ECG signal processing due to its capability to analyze non-stationary signals. The WT involves breaking down the signal into a collection of functions derived from a single function referred to as the mother wavelet, achieved through dilations, contractions (scaling) and time shifts of this function [20]. The CWT entails convolving a signal *x*(*t*) with a wavelet function *ψ*(*t*) that is translated in time by a parameter *b* and scaled by a parameter *a*. The mathematical representation is as follows:

(1) $$CWTx\;\left(a,b\right)=\frac{1}{\sqrt{a}}\int_{-\infty }^{\infty }x\left(t\right)\;\psi \left(\frac{t-b}{a}\right)\;dt$$

One drawback of the implementation of the CWT is that the parameters *a* and *b* are usually discretized, and the analysis is restricted to scales that are powers of two obtaining the dyadic wavelet transform that can be computed with Mallat’s algorithm [20]. Because of this limitation, the implementation of CWT with splines allows a finer discretization of the CWT at the integers *a* y *b* so that it can handle more scales [21].

#### 2.1.2. Modules for Obtaining the Wavelet Transform with Splines

To compute the continuous wavelet transform (CWT) at any integer scale, we employed the *B*-spline-based method introduced by Unser et al. [21]. The mother wavelet used is the first derivative of a cubic *B*-spline of order 4 expanded by a factor of 2 ([21], Figure 2a). This method consists of three stages; therefore, to facilitate its implementation on the FPGA, each stage has an independent submodule. During the implementation of these submodules, certain issues arose. The first one is that they must provide real-time results, which means they do not have access to all the data simultaneously but rather receive and deliver one data point at a time. This leads us to the second issue: the need for memory to store previously calculated data, as the wavelet transform is not a linear function but depends on the signal’s behavior over time. 

#### 2.1.3. First Submodule

With all the elements mentioned before, the first module is presented, which responds to filtering in the input signal as a result of the convolution of the *B*-spline coefficients with the input signal (Equation (2)).

(2) $$s_{1}:=\langle s\left(x\right),\beta^{n_{2}}\left(\left(x-k\right)/m\right)\rangle =b^{n_{1}+n_{2}+1}*{\left(b^{n_{1}}\right)}^{-1}*s\left[k\right]≅b^{n_{2}}*s\left[k\right]$$

where *s*[*k*] is the discrete ECG signal and $b^{n_{2}}$ are the coefficients for calculating the second-order first derivative of the cubic spline, obtained in [21] and whose values are shown in Table 1.

As shown in Table 1, the coefficients can be expressed as fractions or values smaller than one; this complicates their implementation on an FPGA because it is not possible to perform divisions or multiply by decimal numbers. To address this issue, it was decided to multiply the coefficients by 2^16^ and round the result to integers (see Table 1). While this rounding introduces some perturbation, it is not significant beyond correcting the amplitude by dividing the result by 2^16^.

For its implementation on the FPGA, the calculation of the result of the first module can be summarized as follows (Pseudocode 1):
**Pseudocode 1** Submodule 1s1= b[0] * ADC_input + in_z[0];z[0] = b[1] * ADC_input + z[1];z[1] = b[2] * ADC_input + z[2];z[2] = b[3] * ADC_input + z[3];z[3] = b[4] * ADC_input;

With the defined procedure, the following entity was established:
entity CWT_Submodule_1_Scale_8 is PORT(   CLK     : in STD_LOGIC;   Rst       : in STD_LOGIC;   Inic      : in STD_LOGIC;   ADC_input    : in STD_LOGIC_VECTOR (23 downto 0);    State       : out STD_LOGIC_VECTOR (1 downto 0);   Result_M1   : out STD_LOGIC_VECTOR (23 downto 0)    in_Z0,in_Z1,in_Z2,in_Z3 :   in std_logic_vector (39 downto 0);    out_Z0,out_Z1,out_Z2,out_Z3 :  out std_logic_vector (39 downto 0)); end CWT_Module_1_Scale_8;

Where CLK is the clock signal of the state machine. When Rst = 0, the values of in_Z0 to in_Z3 are set to zero; otherwise, they retain their current values. If Inic = 1, the calculations of Result_M1 and z[0] to z[3] are initiated; otherwise, no action is taken. The input ADC_input represents the digitized ECG signal. The ‘State’ output can take four values:

- ‘00’: When Rst = 0, it indicates that the module is locked.
- ‘01’: When Rst = 1, it indicates that the module is not locked but waits for Inic = 1 to start reading (Inic should remain 1 until the obtained result is read).
- ‘10’: Indicates that the values are being calculated.
- ‘11’: Indicates that the calculation of values is complete.

**Figure 3:**
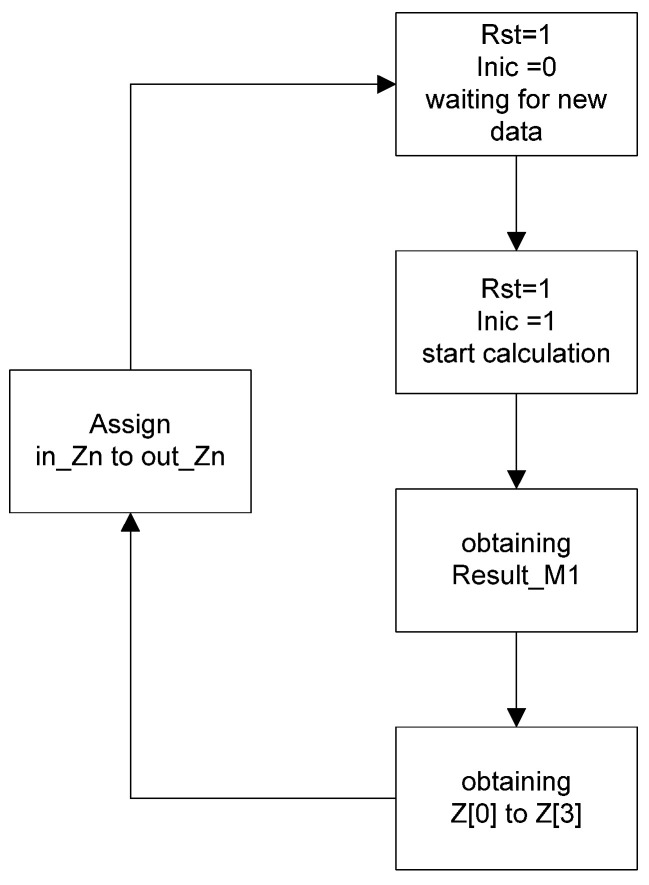
Flowchart of the first submodule.

Finally, the output Result_M1 holds the value that is sent to the next submodule. It is worth noting that for the correct operation of this module, when declaring the signals, it is necessary to connect in_Z0 with out_Z0, and so on up to in_Z3 with out_Z3 (see Figure 3). This connection is essential because the module has a memory effect, relying on previous values. For example, the computation in line 1 of pseudocode 1 would be executed as follows: out_Z0 = b[1] * ADC_input + in_Z1.

#### 2.1.4. Second Submodule

For the second module, used to obtain the CWT, a motion sum filter was implemented as defined by Equation (3):

(3) $$s_{m}:=\langle s\left(x\right),\beta^{n_{2}}(\left(x-k\right)/m)\rangle =u_{m}^{n_{2}}*s_{1}\left(k\right)$$

This filter can be evaluated using two additions per sample using a recursive update strategy:

(4) $$r_{i}\left(k\right)=r_{i}\left(k-1\right)+r_{i-1}\left(k+l_{0}+m\right)-r_{i-1}\left(k+l_{0}\right)$$

where:

- *k* is the position in the input vector.
- $l_{0}$ is the offset (In this work, it takes a value of 0).
- *m* is the scale.

The implementation of the second module is summarized as follows (Pseudocode 2):
**Pseudocode 2** Submodule 2r0(k) = Input_Signalr1(k) = r1(k − 1)+ r0(k + m) − r0(k)r2(k) = r2(k − 1)+ r1(k + m) − r1(k)r3(k) = r3(k − 1)+ r2(k + m) − r2(k)r4(k) = r4(k − 1)+ r3(k + m) − r3(k)r4(0) = r4(1)Output = r4(0)

With the defined procedure, the following entity was established:
entity CWT_Submodule_2_Scala_8 is PORT(   CLK      :  in STD_LOGIC;  Rst         :  in STD_LOGIC;  Inic        :  in STD_LOGIC;  Input_Signal   :  in STD_LOGIC_VECTOR (23 downto 0);  State      :  out STD_LOGIC_VECTOR (1 downto 0);  Result_M2    :  out STD_LOGIC_VECTOR (23 downto 0);
  out_R0_0,…, out_ R0_29  : out STD_LOGIC_VECTOR (30 downto 0);  out_R1_0: out  STD_LOGIC_VECTOR (30 downto 0);  out_R2_0: out  STD_LOGIC_VECTOR (30 downto 0);   out_R3_0: out  STD_LOGIC_VECTOR (30 downto 0);   out_R4_0: out  STD_LOGIC_VECTOR (30 downto 0);    in_R0_0,…, in_R0_29   : in STD_LOGIC_VECTOR (30 downto 0);   in_R1_0: in STD_LOGIC_VECTOR (30 downto 0);  in_ R2_0: in STD_LOGIC_VECTOR (30 downto 0);  in_ R3_0: in STD_LOGIC_VECTOR (30 downto 0);  in_ R4_0: in STD_LOGIC_VECTOR (30 downto 0);  Resultado_M2   :  out STD_LOGIC_VECTOR (23 downto 0)); end CWT_Submodule_2_Scale_8 

**Figure 4:**
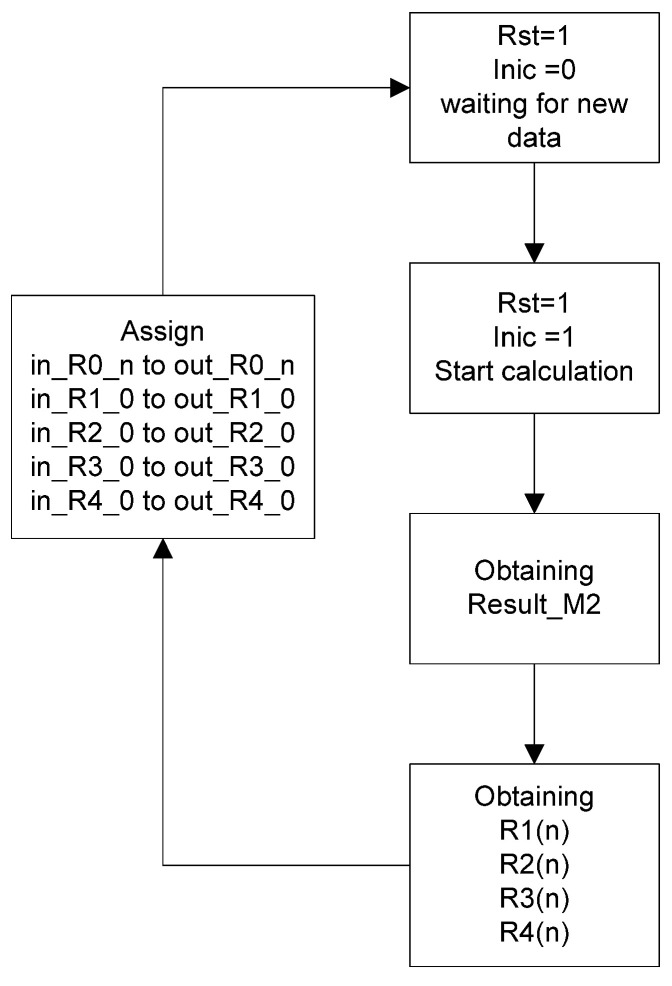
Flowchart of the second submodule.

For this submodule, the behavior of CLK, Inic, and State is the same as in the first submodule. In the case of the Input_Signal, it receives the result from module 1, and Result_M2 is the result of the calculation. As for the rest of the inputs or outputs, they are externally interconnected when the module is declared again due to the memory effect, which relies on previous values. In this case, only the values of R0 are retained because it acts as the memory for the data obtained from module 1, and the first calculated values from R1 to R4 are also preserved, as they become R1_0, R2_0, R3_0 and R4_0 in the subsequent calculation, unlike the rest, and are not recalculated (see Figure 4).

#### 2.1.5. Third Submodule

The third module for obtaining the CWT involves filtering with the *B*-spline coefficients of the wavelet *ψ*(x), as defined in Equation (5):

(5) $$w_{m}\left(k\right)={\left[p\right]}_{↑m}*s_{m}\left(k\right)$$

The FIR operator “*p*” is characterized as a vector of size “n_p_” obtained from ([21], Table 1). In this work, a cubic expanded wavelet spline function with a factor of two was used ([21], Figure 2a), and its coefficients are shown below:

$$p_{1}^{\left(2\right)}=\left(-1,-4,-5,\;0,+5,+4,+1\right)$$

This vector is scale-dependent; therefore, it is rewritten as follows:

(6) $$p_{m}^{\left(2\right)}=\left(-1,z_{m-1},-4,z_{m-1},-5,z_{m-1},0,z_{m-1},+5,z_{m-1},+4,z_{m-1},+1\right)$$

where z*_m_*_−1_ represents the number of zeros added based on the scale; for example, for m = 3, “*p*” would be as follows:

$$p_{3}^{\left(2\right)}=\left(-1,0,0,-4,0,0,-5,0,0,0,0,0,+5,0,0,+4,0,0,+1\right)$$

**Figure 5:**
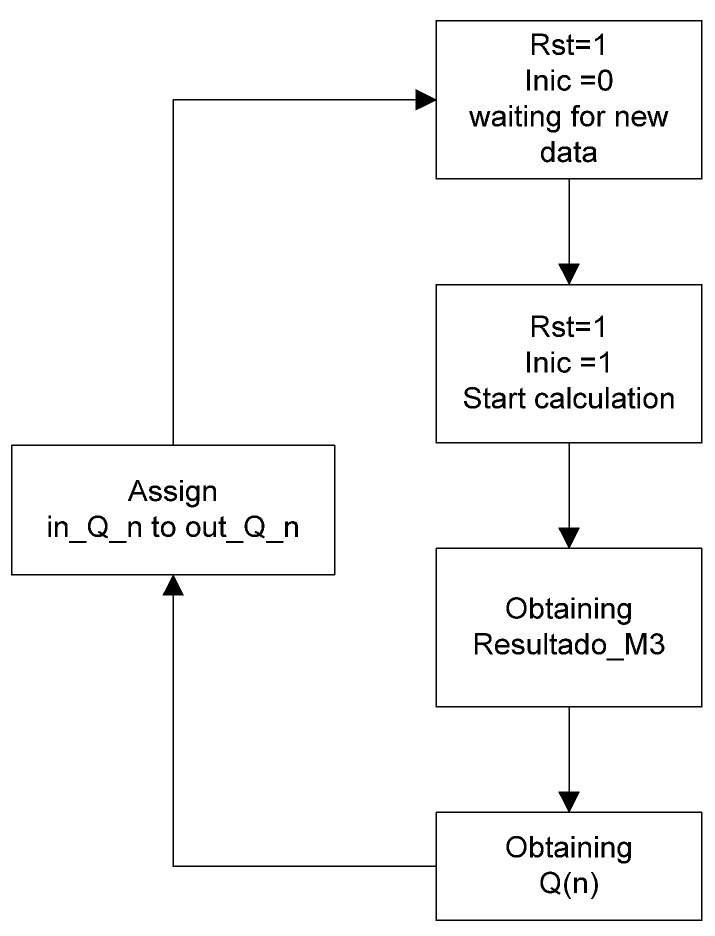
Flowchart of the third submodule.

Once the method of obtaining “*p*” was defined, we established how to calculate the output signal of the CWT in the third submodule (Pseudocode 3):
**Pseudocode 3** Submodule 3Result_M3= p[0] * Input_Signal + Q[0]; Q[0–6] = Q[1–7]; Q[7] = p[1] * Input_Signal + Q[8]; Q[8–14] = Q[9–15]; Q[15] = p[2] * Input_Signal + Q[16]; Q[16–22] = Q[17–23]; Q[23] = p[3] * Input_Signal + Q[24]; Q[24–30] = z[25–31]; Q[31] = p[4] * Input_Signal + Q[32]; Q[32–38] = Q[33–39]; Q[39] = p[5] * Input_Signal + Q[40]; Q[40–46] = Q[41–47]; Q[47] = p[6] * Input_Signal;

With the defined procedure, the following entity was established:
entity CWT_Submodule_3_Scale_8 is PORT(    CLK     : in STD_LOGIC;   Rst       : in STD_LOGIC;    Inic      : in STD_LOGIC;    Input_Signal  : in STD_LOGIC_VECTOR (23 downto 0);    Estado    : out STD_LOGIC_VECTOR (1 downto 0);    Result_M3   : out STD_LOGIC_VECTOR (23 downto 0)); in_Q0,…,in_Q47    : in  std_logic_vector (27 downto 0); out_Q0,...,out_Q47: out  std_logic_vector (27 downto 0); end CWT_Submodule_3_Scale_8;

In this submodule, the behavior of CLK, Inic and Estado is the same as in modules 1 and 2. For the Input_Signal, it receives the result from submodule 2, and Result_M3 holds the result of the CWT calculation. The inputs and outputs Q0 to Q47 are externally interconnected when the module is declared due to the memory effect, which relies on previous values (see Figure 5).

Once CWT is obtained data by data, it is necessary to create a state machine that detects both the ECG points R and Te in order to calculate the heart rate and RT interval.

#### 2.1.6. R wave Detection State Machine

**Figure 6:**
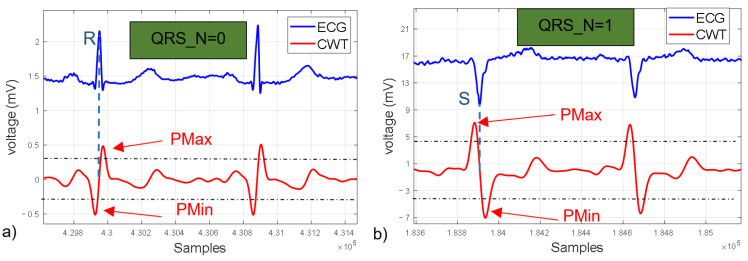
Polarity of QRS complex and its CWT. (**a**) QRS with positive polarity. (**b**) QRS with negative polarity.

**Figure 7:**
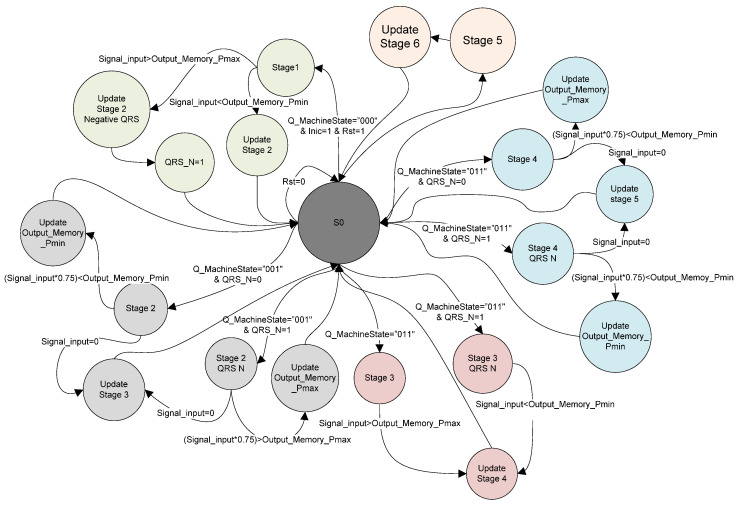
State machine for R wave detection for 2 QRS complex morphologies.

An important characteristic of the ECG is that its waveform varies depending on the lead used, which also modifies the waveform of the CWT (Figure 6). This work is based on the algorithm proposed by Alvarado et al. [22] and the VHDL module for detecting the R wave by Martínez et al. [23]. Both methods consist of detecting a negative lobe (Pmax), followed by a positive lobe (Pmin), and the zero-crossing between these two points corresponds to the R wave peak (Figure 6a). However, this method is limited to only one QRS complex morphology (positive R wave), as other leads such as aVR, VI, V2 and DIII tend to have a negative polarity (Figure 6b). To address this, a modification is proposed that includes a flag (QRS_N) that allows the algorithm to adapt and detect different morphologies based on which lobe is detected first (Pmin or Pmax). Detecting the R wave is important as it serves as a starting point for identifying other waves or points in the ECG, such as Te in this case. For this purpose, a state machine was implemented for R wave detection based on the QRS_N flag (Figure 7).

The first stage is responsible for detecting either the Pmax or Pmin point in the CWT. If the value received by the modules to obtain the CWT “Signal_Input” is greater than the value stored in the variable ‘Output_Memory_Pmax’, the QRS_N signal takes the value of 1, indicating a possible QRS with negative polarity. On the other hand, if the value of “Signal_Input” is lower compared to the value stored in the variable ‘Output_Memory_Pmin’, the QRS_N signal remains with its initial value of zero.

The second stage is subdivided into three functions:

- The first function detects if “Signal_input” multiplied by 0.75 is greater than “Output_Memory_Pmax” or less than “Output_Memory_Pmin”, depending on the value of QRS_N, and updates its value.
- The second function is responsible for detecting the zero-crossing of the CWT (peak of the R wave).

- The third function stores the value of the time counter (RR Counter) in the variable “OutputSignalQRS” when the zero-crossing is detected and resets the counter. It should be noted that in this function, the counter is reset for measuring the RT interval.

The third stage is responsible for detecting the point, either Pmax or Pmin (depending on the value of QRS_N), by comparing the “Signal_input” value with the value stored in the variable “Output_Memory_Pmin” or “Output_Memory_Pmax”, as appropriate.

The fourth stage is divided into two functions:

- The first function detects if the value of Signal_input modules multiplied by 0.75 is greater than “Output_Memory_Pmax” or less than “Output_Memory_Pmin”, depending on the value of QRS_N, and updates its value.
- The second function is responsible for detecting a second zero-crossing.

The fifth stage introduces a delay of 40 ms before starting the search for the point Te.

One challenge faced by ambulatory ECG monitoring systems is the variation in the ECG signal amplitude. This variation occurs due to different factors, with one notable cause being the degradation of the electrode–skin interface over time, as the conductive gel dries out. As a result, the values of “Output_Memory_Pmax” and “Output_Memory_Pmin” cannot be fixed. To address this issue, the algorithm is designed to adapt. Initially, the variables “Output_Memory_Pmax” and “Output_Memory_Pmin” have a value of zero, and during the first 60 s of acquisition, the algorithm updates these values in stages 2 and 4 (the initial 60 s of QRS complex detection are not reliable).

Another challenge in ECG acquisition is the presence of noise due to imperfect mechanical contact in the electrode–skin interface, which can cause “Output_Memory_Pmax” and “Output_Memory_Pmin” values to spike, preventing the detection of Pmax and Pmin points. To address this, a protection mechanism was developed. If more than 3 s pass without detecting the Pmin or Pmax point (QRS_N = 1), the values of “Output_Memory_Pmax” and “Output_Memory_Pmin” are halved until reaching zero, and the state machine returns to the first stage.

#### 2.1.7. Te Detection State Machine

**Figure 8:**
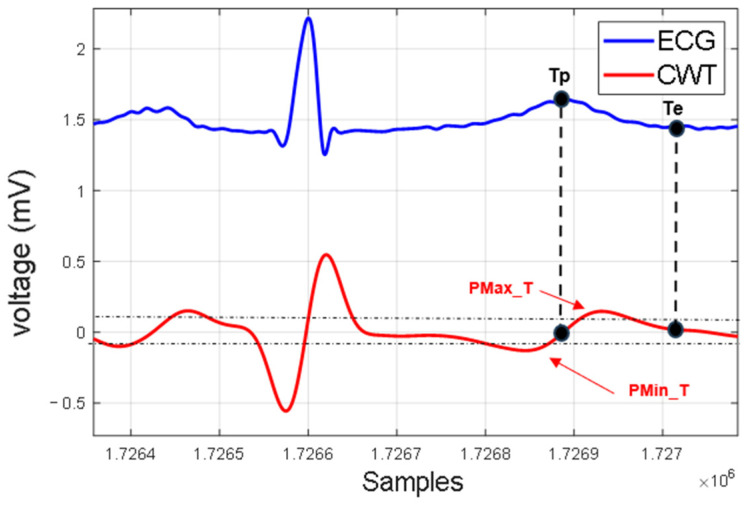
Characteristic points Tp and Te of T wave and its CWT.

**Figure 9:**
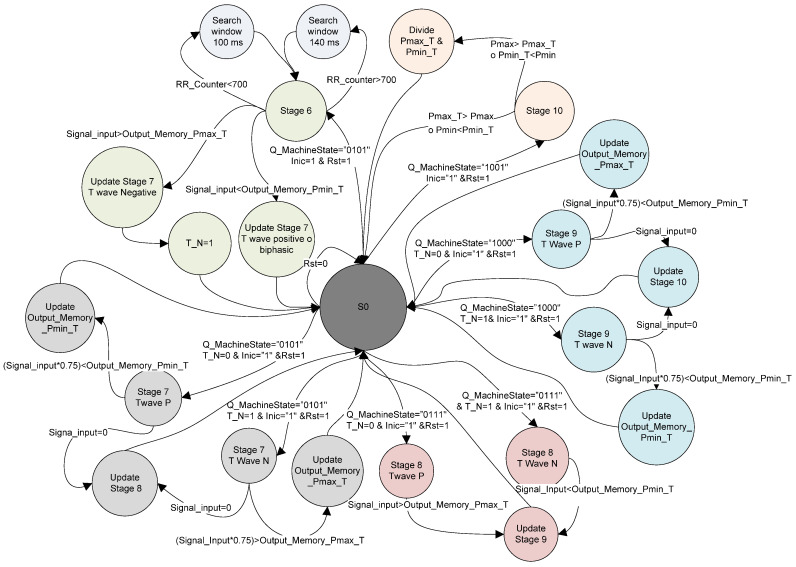
State machine for Te detection for 2 T wave morphologies.

The advantage of using scale 8 of the CWT is that it significantly enhances the T wave, as observed in Figure 6, allowing for resource savings by avoiding the implementation of an additional scale in parallel. Unlike the established method for detecting the R wave, detecting the T wave does not involve finding the zero-crossing between positive and negative lobes. Instead, the goal is to find the zero-crossing after the two lobes, corresponding to the point Tp of the T wave, as shown in Figure 8. Detecting Te requires a similar structure to the QRS complex, a state machine capable of detecting variations in the T wave’s shape (polarity, positive, negative and biphasic), which led to the implementation of 5 additional fundamental states (Figure 9).

The sixth stage is divided into two functions:

- The first function defines a search window that depends on the last value of “RR_counter”. If the condition (RR_counter > 700 ms) is met, the search window for the first maximum or minimum of the CWT will be 140 ms. Alternatively, if (RR_Counter < 700 ms), the search window is set to 100 ms.
- The second function is responsible for detecting either the “Pmax_T” or “Pmin_T” point by comparing the value of “Signal_input” with the value stored in the variable “Output_Memory_Pmax_T” or “Output_Memory_Pmin_T”, respectively. If the input signal is greater than the “ Output_Memory_Pmax_T” variable, then the “T_N” signal is set to one; otherwise, it remains at its initial value of zero.

The seventh stage is divided into two functions:

- The first function detects if the value of “Signal_input” multiplied by 0.75 is greater than “Output_Memory_Pmax_T” (for “T_N = 1) or, conversely, if it is less than “Output_Memory _Pmin_T” (for “T_N = 0”). If the condition is met, the corresponding variables are updated.
- The second function detects the first zero-crossing.

The eighth stage is responsible for detecting either the Pmax_T or Pmin_T point, depending on the value of the “T_N” variable.

The ninth stage is divided into three functions:

- The first function detects if the value of “Signal_input” multiplied by 0.75 is greater than “Output_Memory_Pmin_T” (for “T_N = 1) or, conversely, if it is less than “Output_Memory_Pmax_T” (for “T_N = 0”). If the condition is met, the variables “Output_Memory_Pmax_T” or “Output_Memory_Pmin_T” are updated, respectively.
- The second function detects the second zero-crossing, which corresponds to the point Te.
- The third function copies the value of the time counter “RT_Counter” to the variable “OutputSignalRT” and resets the counter.

The tenth stage compares the maximum and minimum values of both the T wave and the QRS complex. If the value of “Output_Memory_Pmax_T” is greater than “Output_Memory_Pmax”, the signal “Output_Memory_Pmax_T” is divided by 2. The same happens with the values of “Output_Memory_Pmin_T” and “Output_Memory_Pmin”. The tenth stage is necessary to avoid confusion between the detection of the QRS complex and the T wave.

## 3. Results and Discussion

The performance of the detection algorithm of R and Te of the ambulatory long-term ECG monitor was evaluated with 10 records of 15 min with different morphologies from the manually annotated QT database (QTDB) [24]. The accuracy for R wave detection was of 97.8% in 11,071 analyzed beats ([19], Table 1). The measurement error for Te was of 5.14 ± 7.07 ms ([19], Table 2), which was within the tolerance limits of standard deviations compared to the manual measurements performed by the CSE experts [25].

**Figure 10:**
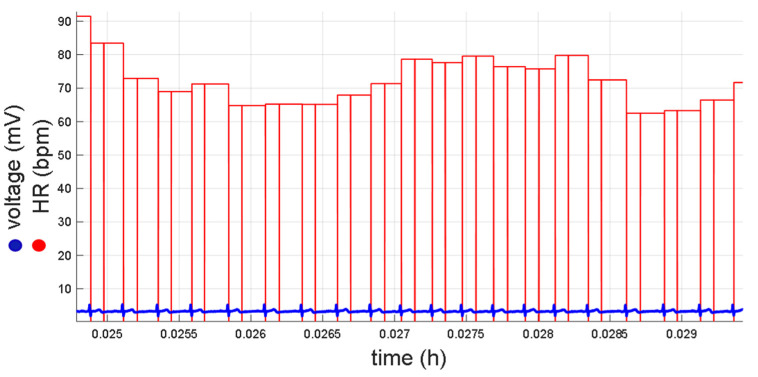
ECG signal (blue) along with the heart rate values obtained from a test subject, and the QRS complex and the Te positions (red).

In order to save storage space on the micro-SD memory, the RT interval values were indirectly stored in HR records, since the system stores the value of zero in the presence of a QRS complex and the Te (Figure 10).

**Figure 11:**
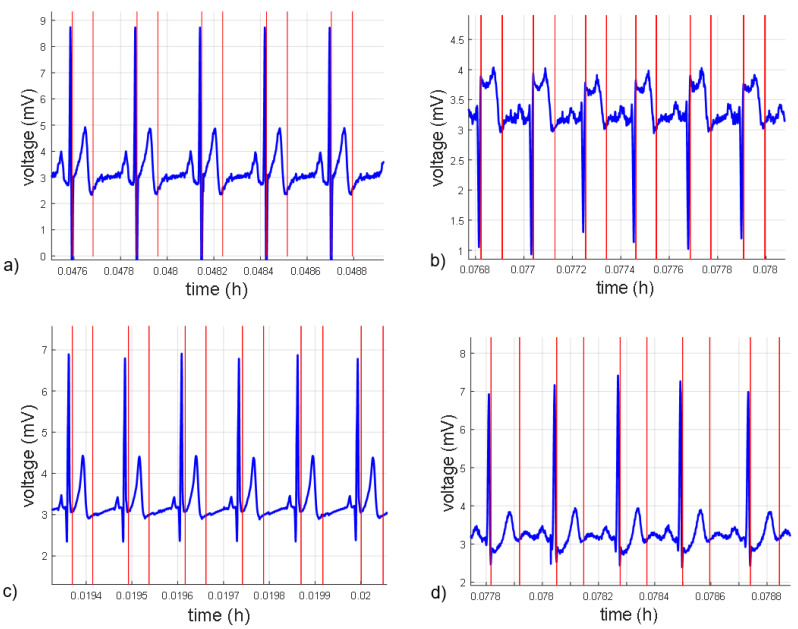
ECG signals (blue) and QRS complex and Te point (red) detections from different records. (**a**) Own record. (**b**) Record nsr001 from the NSRDB. (**c**) Record nsr001 from TWADB. (**d**) Record se103 from QTDB.

Since the long-duration recordings were obtained from normal subjects, those where the ECG signal had a T wave of high amplitude or a QRS complex with negative polarity were selected. Figure 11 presents the results obtained in the detection of QRS complex and Te for the measurement of RT interval from an own record and representative records of three databases: QTDB [24], T-Wave Alternans Challenge Database (TWADB) [26,27], and the Normal Sinus Rhythm RR Interval Database (NSRDB) [27].

However, as can be seen (Figure 11), a slight delay of 36 samples was found in the detection of the points when using a scale of 8 and a sampling frequency of 1 KHz, which is equivalent to a delay of 36 ms. This delay is caused by the calculation of the CWT; however, it does not affect the values of HR and the RT interval, as both use the same scale. Furthermore, the delay is constant, allowing for compensation offline.

**Figure 12:**
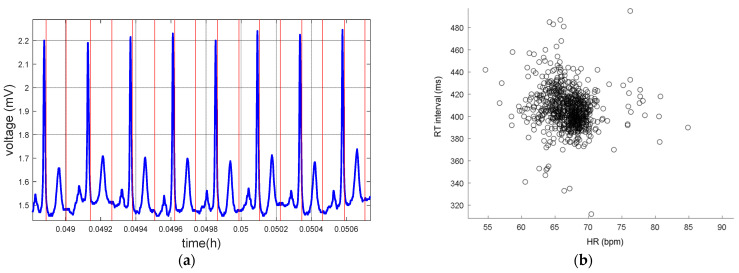
Record sel30 from QTDB. (**a**) ECG signals (blue) and QRS complex and Te point (red) detections. (**b**) Dynamics RT-HR.

**Figure 13:**
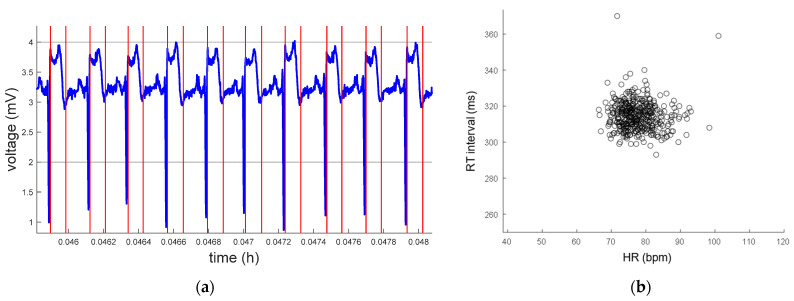
Record nsr001 from NSRDB. (**a**) ECG signals (blue) and QRS complex and Te point (red) detections. (**b**) Dynamics RT-HR.

Figure 12 and Figure 13 show the detections of QRS complex and Te, and the dynamics of the relationship between RT interval and HR in recordings from QTDB and NSRDB.

**Figure 14:**
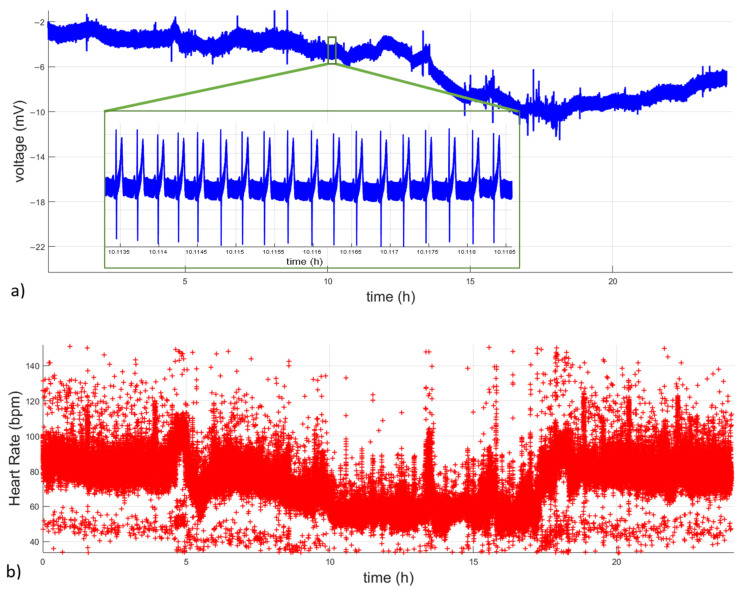
Long-term recording of ECG acquired by the prototype of an ambulatory ECG monitor. (**a**) ECG signal. (**b**) HR.

Figure 14 shows a long-term recording obtained with the Holter prototype, displaying the ECG signal and HR values. It is possible to observe the presence of some motion artifacts during daily activities in certain parts of the recording.

## 4. Conclusions

In this work, we have presented the implementation in an FPGA of real-time detection algorithms of QRS and Te based on the CWT with splines for the beat-to-beat measurements of heart rate and RT interval of a prototype of an ambulatory long-term ECG monitor of three leads.

The algorithms had good performance in the 10 records of 15 min with different morphologies of QRS complex and T wave from the validation QT database. An accuracy of 97.8% in 11071 analyzed beats was obtained for QRS complex detection. For Te detection the measurement error was of 5.14 ± 7.07 ms, which is within the tolerance limits for deviations with respect of the manual measurements by CSE experts.

An offline analysis for the QRS complex and Te detection involves using two different scales (low and high) of the CWT, which, when implemented in an FPGA, increases the computational cost and complexity due to the synchronization of processes with different delays. The designed algorithm has an implicit advantage by using only one scale of the CWT to detect QRS and Te, significantly reducing the computational cost in the FPGA. The implementation of the CWT in the FPGA generates a delay that is a function of the scale used and the sampling rate, but the delay is a constant delay that does not affect the values of the heart rate and the RT interval.

The use of state machines provides flexibility to detect variations in the morphology of the ECG, both in the QRS complex (positive and negative polarity) and in the T-wave (positive, negative and biphasic), whether in the analyzed lead or in the presence of pathologies. Changes in T wave morphology can be detected indirectly by calculating the RT interval dispersion (difference between the maximum and minimum RT intervals in the three leads), since previous studies have shown that these changes are related. In future work, it is suggested to analyze more specific morphology variations, such as the presence of ectopic beats with subsequent RR interval changes for the analysis of parameters with predictive value of cardiovascular death and arrhythmic events as the heart rate turbulence. Furthermore, adding an algorithm to detect the T wave peak to measure the RTpeak and Tpeak-Tend intervals that are considered as risk predictors of arrhythmic events is recommended.

## Figures and Tables

**Table 1:** Filter coefficients implemented before and after modification.

	Original Coefficients	Coefficients Multiplied by 2^16^
b0	1/120 = 0.0083	543
b1	1/26 = 0.2167	14,201
b2	1/66 = 0.5500	36,044
b3	1/26 = 0.2167	14,201
b4	1/120 = 0.0083	543

## Data Availability

All the necessary data are included in the article.
